# Clinical Lectures on the Principles and Practice of Medicine

**Published:** 1858-07

**Authors:** 


ART. III.—*Clinical Lectures on the Principles and Practice of Medicine.* By JOHN HUGHES BENNETT, M.D., Professor of Institutes of Medicine and of Clinical Medicine in the University of Edinburgh, etc. New York: Samuel S. & William Wood. Second edition; 8vo., pp. 951. 1858.*

---

* This work is the English edition with an American title-page, and is an admirable specimen of a well printed and illustrated book. If this practice of thus importing books in large numbers were more followed out, we might be saved from the miserable reprints with which we are annually flooded.

FEW of the present generation have evinced a more lively zeal for the promotion of medical science than the author of the work before us; and yet fewer have had their names coupled with so many discoveries which have advanced its boundaries, and have led to more important results. The discoverer of leucocythæmia, the introducer, or, at least, the diffuser of the knowledge of the beneficial effects of cod-liver oil, may lay claim to have been a laborer alike in the field of science and in that of humanity, and to have gleaned flowers whose brightness no scorching breath of envy, nor stormy change of doctrine, can wither or destroy. In this volume much of the research is for the first time collected, and the doctrines which have been gradually formed, are clearly announced and skillfully defended. This gives to these Lectures much more of a controversial character than works on Clinical Medicine usually possess. Whether this be an advantage or not, every reader will decide according to his individual opinion.

Dr. Bennett commences his work with some chapters which, although interesting, can yet hardly be construed to belong to the subject of Clinical Medicine: such as the relation of the science to the art of medicine; the political state of the medical profession; its social state. Indeed, throughout, we cannot avoid being struck with the fact that much matter is introduced which the title-page by no means calls for, and would seem to forbid. The reader will find this illustrated as we proceed in our analytical task.

The chapter on the present state of practical medicine embraces some remarks which cannot be allowed to pass unnoticed, as they explain much of the scope and aim of these Lectures.

"Although seventeen years have elapsed since the cell doctrine of growth has been admitted into physiology and pathology, medical men have not yet realized to themselves its vast importance in a practical point of view. The morbid processes of inflammation, of tuberculization, and of various morbid growths, are now for the most part elucidated by this theory. But a cell pathology is no more universally applicable to the phenomena of disease than is humoralism or solidism. Indeed, we may more correctly speak of a molecular pathology, as a molecule, and not a cell, is the first and last form of organization. What, however, it is important to remember here is, that if there be a molecular or a cell physiology and pathology, so is there a molecular and a cell therapeutics. For, it is evident, that those diseases which depend on an increase or diminution of cells, can only be reached scientifically through a knowledge of those laws which govern their evolution and disintegration.

"Thus, growth (that is, the multiplication of cells) is favored by increased warmth, by room for expansion, and by moisture;—and it is checked by cold, by pressure, and by dryness. If, then, an exudation be poured out and coagulated near the surface, as it can only disappear by its passing through the stages of cell growth, we favor suppuration, that is, the growth of pus cells, by warm poultices or fomentations, and retard it by cold and pressure."

These remarks are followed by a brief but able eulogy on the more modern means of investigation,—the microscope, auscultation, histological and chemical analyses. To them, in truth, must we look for the future constant and steady development of medicine : to them to that scrutiny which shall teach us to simplify our precepts, and to bring our practice in connection with the grand fundamental laws which govern every organism. But the time for the substitution of absolute laws (gained by our modern research) for those derived from empirical observation, has not arrived; so that when our talented author speaks of the "wreck of ancient systems," and the "approaching downfall of empirical practice," we respect his enthusiasm, we sympathize with his longing for determinate, fixed principles, uniting the laws of health and disease; but we also think of the years of unwearied investigation, the furrows that will deepen many a now youthful brow, the generations that will pass away and arise, before so many needful observations and carefully-ascertained facts shall be arranged into a harmonious whole, and be ready to be placed into the crucible, to have the pure glistening element of fundamental truth extracted.

The general method of examining a patient, and some of the more special modes of investigating disease, are ably described in the first section of the work.

The general examination is conducted on a similar plan to that followed by Professor Rostan in his ward at Hôtel Dieu, and is certainly exceedingly careful and minute. Let us take the inquiry into the nervous system, and into the digestive system as its type.

"NERVOUS SYSTEM.—*Brain*—Intelligence—augmented, perverted, or diminished; cephalalgia; hallucinations; delirium, stupidity, monomania, idiocy; sleep, dreams, vertigo, stupor, coma. *Spinal cord and nerves*—Pain in back; general sensibility, increased, diminished, or absent; special sensibility—sight, hearing, smell, taste, touch, their increase, perversion, or diminution; spinal irritation, as determined by percussion; motion, natural or perverted, fatigue, pain on movement; trembling, convulsions, contractions, rigidity, paralysis.

"DIGESTIVE SYSTEM.—*Mouth*—Lips, teeth, and gums; taste in the mouth. *Tongue*—Mode of protrusion, color, furred, coated, fissured, condition of papillæ, moist or dry. *Fauces, tonsils, pharynx, and œsophagus*—Deglutition—if impeded, examine the pharynx with a spatula, the cervical glands, neck, etc.; regurgitation. *Stomach*—Appetite, thirst, epigastric uneasiness or pain, swelling, nausea, vomiting, character of matters vomited, flatulence, eructations. *Abdomen*—Its measurement and palpation; pain, distension, or collapse, borborygmi, tumors, constipation, diarrhœa, character of dejections, hemorrhoids. *Liver*—Size, as determined by percussion, pain, jaundice, results of palpation, etc. *Spleen* —Size, as determined by percussion. If enlarged, examine blood microscopically."

The chapter on the special modes of investigation by palpation, mensuration and percussion, contain a brief summary of the facts connected with these methods of physical diagnosis; we would merely notice, in passing, that Dr. Bennett approves strongly of an ivory pleximeter and of a percussion hammer to elicit clear tones. So impressed is he with their superiority, that he states it as his belief that by means of these instruments, after an hour's practice on a dead body, the student is placed on a par (as regards the art of percussion,) with the generality of English practitioners; which statement any one who has used the instruments may well take the liberty of questioning, if he does not wish to be forced into the inference that the English practitioners possess no skill at all. Another special mode of investigation, the microscope, has its claims as a clinical aid more convincingly demonstrated; and if there be any of our readers who are still inclined to ask that superfluous question, What practical results has the microscope achieved? we may point, as illustrating its powerful aid in diagnosis, to the examples cited by Dr. Bennett. Let us take one among many:—

"An elderly lady conceived herself to be affected with insects continually forming in the skin, which produced incessant itching and tingling. All the hair was removed, and every kind of application, including mercurial preparations, were tried without effect. On rubbing the surface she always saw minute white rolls and black specks, which she regarded as insects in different stages of development. The torment and anxiety this caused her for many months it is scarcely possible to conceive. At length she labored under the idea that she was communicating the disease to her husband and daughter, when, at the request of her medical attendant in the west of Scotland, she came to Edinburgh, in order that I might investigate and treat it. I had the pleasure of showing this lady, under the microscope, that the white bodies were minute rolls of epidermis, or of the cotton cloth with which she rubbed the skin, and that the black specks were portions of dust or soot. Her hallucination being in this way dissipated, she returned home perfectly well."

In a clinical examination of the blood, of the sputum, the milk, and the secretions generally, we possess in the microscope the surest and most reliable, we might say the only reliable method of arriving at accurate results. These subjects are here most appropriately discussed, and the different minute appearances of secretions and discharges well delineated; we except Fig. 73, representing urate of ammonia, the granules of which are, to our eye, entirely too large and out of proportion.

The second section of the work, treating of the Principles of Medicine, commences with several chapters which more properly belong to the Institutes of Medicine. Thus, we have a chapter on the General Anatomy and Physiology of the Nervous System, in which cognizance is taken of the investigations of Owsjannikow on the minute structure of the spinal marrow. It is followed by some remarks on the general pathology of the nervous system, maintaining the old doctrine of Monro of the amount of fluid within the cranium always being the same. Dr. Burrow's researches on the subject, and their explanation, are therefore not adopted. But while, of course, we cannot find fault with this, we must object to the statement that by a misconception of the older views on the part of Dr. Burrows, he has only contrived to overthrow a doctrine of his own creation. The doctrine he attempted to overthrow was certainly the one (until recently) very prevalent and usually adopted, that there was always an equal amount of blood in the brain. It was a doctrine which had been sanctioned by Abercrombie, Kellie, Clutterbuck, and Watson. This, at least, we think any candid reader of their recorded opinions will believe them to have expressed. Now, Dr. Bennett maintains that they meant *fluid*, and not *blood*. With reference to the prevalent opinion, and to that of the distinguished men cited, we cannot admit this, although we think that Monro, the originator of the whole theory, may have possibly entertained some such view, as the following passage will prove:—

"For, as the substance of the brain, like that of the other solids of our body, is nearly incompressible, the quantity of blood within the head must be the same, or very nearly the same, at all times, whether in health or disease, in life or after death, those cases only excepted in which water or other matter is effused or secreted from the blood-vessels, for in these a quantity of blood, equal in bulk to the effused matter, will be pressed out of the cranium."—*Observations on Structure and Functions of the Nervous System*, Edinburgh, 1783.

The study of *exudations* is of such importance, and comparatively so identified with the progress of modern medicine, that we looked with great interest to this portion of Dr. Bennett's work. And we have not been disappointed. Here and there may be a few statements to which exception might be taken; but a more concise and masterly exposition of these important points of pathology it would hardly be possible to find.

Exudations are divided into simple,—as they occur on serous membranes, on mucous membranes, or in areolar tissue, or in dense parenchymatous organs, and also simple exudations, as seen in granulating surfaces,—into cancerous, and tubercular exudations. In the last there is no disposition to perfect cell formation, but much to disintegration. The cancerous exudation is distinguished by its rapidly developing corpuscles and its tendency to organize a perfect stroma; while the first form may break down into pus or change into fibres, whose further development, if active, leads to permanent bands and growths. Taking, to use the language of our author, the products of simple exudation as a standard, we cannot fail to remark that, while the cell development of tubercle is below, that of cancer is above this standard; of the three kinds of exudations tubercle is the lowest, and cancer the highest in the scale.

What the ultimate cause is which produces this difference in the formative power of the exudation we know not. Dr. Bennett agrees with the majority of pathologists in ascribing the most important part to the diversities in the composition of the blood. But he also suggests that the elements which determine the constitution of the blood, as the digestion in the alimentary canal and the secondary digestion in the tissues, should be more carefully studied in reference to the different states of the exudations, since these being derived from the blood, the causes which determine the composition of this vital fluid must also possess a most important influence in determining the variety of exudation. All the primary morbid growths, themselves the development of the exudations, are ranged under nine heads:—fibrous growths; fatty growths; vascular growths; cystic growths; glandular growths; epithelial growths; cartilaginous growths; osseous growths; cancerous growths. They are well described, and illustrated with admirable wood-cuts; so, too, are the morbid degenerations of texture and the concretions. The latter embrace urinary calculi; a subject which it is not usual to discuss in works on clinical medicine. With regard, further, to many of the above-mentioned growths this remark will hold good.

The more correct views held with reference to most morbid processes have doubtlessly led to changes in our treatment of disease. Dr. Bennett believes that the great difference between our present therapeutics and those of former days is *alone* attributable to the more intimate knowledge of disease. That the advance of medicine, both in ascertained facts and in correct application of principles, within the last twenty years has been unparalleled, no educated physician will for one moment deny. This advance is our pride. Who will gainsay that the careful examination of morbid processes and tissues has taught us much of the natural history of disease, and has cured much of the evil of overdosing? Who does not feel grateful at this result? But whether there has not also been an intrinsic change in disease, thus calling for different remedies, is a question which first requires to be solved, and which has involved our author in a heated controversy, carried on by able argument and industry by him, as well as by his noted opponents. He sums up here in his lectures much of the ground he has been over in his statements in medical journals; and a question of such vital importance to the profession we feel ourselves bound to touch upon, regretting that the necessary limits of a review will forbid us from following the arguments in detail.

But let Dr. Bennett state his own case:—

"Even to enumerate the changes which have recently taken place in medical practice, as a result of this mode of viewing diseases, would be here impossible. They will be referred to in the special part of the work. Two great facts, however, seem to me, from their universal importance, to demand attention at present. These are—1st. The diminished employment of blood-letting and other antiphlogistic remedies in the treatment of acute exudations or so-called inflammations. 2d. The power which it has been demonstrated may be exercised over certain diseases of innervation, through the influence of suggestion or strong impressions made upon the mind. Both these facts have recently excited great attention and discussion; their influence on medical theory and practice has already been great, and their explanation on scientific grounds seems to be called for with a view of establishing correct principles for our future guidance."

The second of these facts we will not further dwell upon; the first Dr. Bennett discusses, to illustrate the view already mentioned, in five propositions, viz.: 1st. That little reliance can be placed on the experience of those who, like Cullen and Gregory, were unacquainted with the nature and mode of detecting internal inflammations. 2d. That inflammation is the same now as it has ever been, and that the analogy sought to be established between it and the varying types of essential fevers is fallacious. 3d. That the principles on which blood-letting and antiphlogistic remedies have hitherto been practiced are opposed to a sound pathology. 4th. That an inflammation once established cannot be cut short, and that the only object of judicious medical practice is to conduct it to a favorable termination. 5th. That all positive knowledge of the experience of the past, as well as the more exact observation of the present day, alike establishes the truth of the preceding propositions as guides for the future.

The first of these propositions, Dr. Bennett argues, is proved by the fact that the former methods of recognizing inflammation were imperfect. The older physicians did not know that exudations of lymph and the diseased condition of a part constituted inflammation. Groups of symptoms "in accordance with the nosological systems of the day constituted inflammation; hence we are not to be guided by their practice." Such is his reasoning. But may it not be said, on the other hand, that although they did not understand the morbid anatomy of inflammation, yet their views of the symptoms accompanying it, derived from experience, could not all have been unreliable? They may not have understood as much as we do of the means of detecting inflammation; they may not have been able to have determined its exact seat; their knowledge of the minute changes was less. But who can affirm that they altogether overlooked most internal inflammations? who can say that they were so blinded as not to see that their therapeutics were never followed by good results; who can establish that these men, so distinguished by their sagacity, should have obstinately closed their eyes to the teachings of common sense? Dr. Bennett will not admit that the internal inflammations they treated were not injuriously influenced by the lancet, while at present many are. His proposition is, that inflammation is the same now as it has ever been. It terminates in effusion of lymph. "How can it be shown," he asks, "that any of these necessary changes have of late years undergone any modification? Are the effects which followed wounds received at the battle of the Alma different from those which resulted from similar injuries at the battle of Waterloo? Again, if a healthy individual now-a-days be exposed to cold or wet, and be seized with an inflammation of the lungs or pleura, is not the lung hepatized in the one case, and do not layers of organizable lymph form in the other in exactly the same way as formerly? If so, is not hepatization removed, and does not the lymph contract adhesions in the same manner now as in the days of Cullen and Gregory?" Now, granting that the essential local features of inflammation are the same, that exudation occurs now as it did then, it does not follow that the state of system is always the same. We see in different sections of this wide country pneumonia prevailing in such different forms, with such opposite symptoms, that although we cannot question that in all exudation takes place, he would be a bold man who would venture to affirm that the symptoms and state of system accompanying the disease in some parts of the Northern States are exactly the same as that low form of pneumonia so prevalent in some parts of the South. Again, in typhoid fever there are always local lesions, which are probably the same now as they were before the time of Louis; but are all epidemics of typhoid fever alike in the activity of the fever? It seems to us that Dr. Bennett does not sufficiently distinguish between the morbid anatomy and the symptoms of disease, between local and constitutional states.

The argument, too, with reference to wounds received on the field of battle, is contrary to the recorded experience of the most eminent of military surgeons. Larrey tells us expressly, that in some campaigns and under some circumstances wounds healed more quickly, or were accompanied by constitutional symptoms which at other times were not perceived. Even the local effects of injuries were not always the same.

"Les plaies d'armes a feu que nos soldats ont reçues en Syrie, aux extrémitiés superieures compliquées de fracture, sur tout celles de l'humerus, quoique pansées méthodiquement et avec soin, ont presque toutes été suivies d'articulations accidentelles." * * * *

"Il est encore arrivé, dans cette même campagne, que de tres légeres blessures aux épaules, sans lésion des os, ont été suivies, chez presque tous les militaires qui les ont reçues, de paralysie complète ou incomplète du membre correspondant à la blessure, ce qui n'arrivé presque jamais en Europe, à moins que les principaux nerfs ne soient coupés ou désorganisés."—*Larrey: Relation Historique et Chirurgicale de l'Armée d'Orient.*

The analogy derived from the varying type of fever, an argument most ably urged against the proposition of the invariable similarity of inflammation by the venerable Emeritus Professor of Medicine in Edinburgh, Dr. Alison, Dr. Bennett will not admit. But why not? Bleeding was as much practiced for fever as it was for inflammation, and has been more completely abandoned. Why, if it can be proved that the symptoms of fever are at one time activity, at others depression, should it not be admitted that there may be unknown circumstances modifying the symptoms accompanying inflammation? Dr. La Roche, in his work on Yellow Fever, tells us that this disease assumes at times a sthenic, at others an asthenic form; and it is a very interesting feature of this controversy, that a colleague of Dr. Bennett's, teaching in the same hospital, practicing in the same city, has of late* come forward and proved, by notes he had kept of the epidemics, the changes which have taken place in the constitution of fevers in Edinburgh during the last forty years. "During the epidemic fever of 1817–20," says Dr. Christison, "the fever, with such a vivid reaction, demanded some sedative. Blood-letting, the most powerful and certain of all, was resorted to; and such were the good effects apparently obtained with it, that it soon came into universal credit, and was carried to what will very naturally now seem extravagant lengths." In a dissertation, written in 1819, we find that the fever was rarely stopped until twenty to thirty ounces of blood were removed, and much more was occasionally taken with the most beneficial results, ("et aliquando e viro procero, forti, et toroso, tres librae, vel amplius, insigni cum beneficio eductæ sunt.") In a lecture, written in 1835, Dr. Christison says,—"To conclude, in former epidemics of fever in this city, the prevailing type of the disease was inflammatory reaction; in the later epidemics, the prevalent character has been nervous depression. And, accordingly, if free depletion was the principal remedy in the former instance, the main remedy in the latter has been wine, with other stimulants."

This was written by a practicing physician long before this controversy began. Bleeding was abandoned in fever because it was found not to answer, and not because we understand the nature of fevers better; for who can affirm that the pathology of fevers is so well understood now as

* Edinburgh Medical Journal, Jan. 1858.

to have so materially modified our practice? There is no reason why this should not, to some extent, hold good with regard to inflammatory diseases.

But a point arises here which we believe to be more susceptible of proof, and of greater importance in solving this question, than the invoking of a change of type of disease at large. It is this: the recorded experience of nearly all our writers is derived from hospital and city practice. Now, is it irrational to conclude that the different social and other conditions we are now living in, with the continued rack of brain and body, may have so influenced the large masses living in cities or in certain districts as to have rendered active antiphlogistics less well borne? Thus, in some parts, from certain influences, undeniable changes have taken place; they are those from which mostly observations are published. This, we think, may be maintained without believing in a general change of type. The state of system, then, not the disease itself, forbids antiphlogistics, and the state of system is determined by special causes.

How these act, we know not. We are aware that anxiety, grief, hygienic, and atmospheric influences have a powerful influence on individual constitutions. We may go further, and suppose (and thus a general change in the type of disease could be accounted for) that at times, in the history of mankind, there may be causes, known or unknown, which powerfully influence us all in health, and in the way in which disease affects us; that there are, to use the beautiful expression of Dr. Watson, waves of time through which the sthenic and asthenic characters of disease prevail in succession, and that we are at present living amid one of its adynamic phases.

The third and fourth propositions we need not stop to inquire into; for, after all, they are, to a great extent, embraced in the last proposition—how far we can determine from experience what is the value of antiphlogistics in acute inflammations. The question of blood-letting and of antiphlogistics is a clinical question, and their value must be decided on clinical grounds, without any reference to prejudged views of their action. Dr. Bennett has taken pneumonia as his standard, and all his remarks and facts, carefully and admirably ascertained as they are, would certainly tend to prove that this disease, as he meets with it, is better treated without than with bleeding. Without mentioning the antagonistic facts which some of his opponents have published concerning the treatment of pneumonia, we would protest against the whole method of reasoning. We will grant that Dr. Bennett has proved much with reference to pneumonia, and is entitled to thanks for so doing; but he has done nothing more; and we declare against the whole question of the use of antiphlogistics being decided on the ground that they do no good in pneumonia.

It is not right to make one inflammation the standard by which to judge all. Let it be *clinically* ascertained that in inflammation of all organs blood-letting and antiphlogistics are not beneficial, and they will be discarded; but because the lancet is useless in pneumonia its value in other diseases is not impaired. It may not cut short diseases, as has been supposed, but does it follow that it has done and does no good? If we were to set aside all but the remedies that cut short a disease, how many would we have left out of the entire materia medica? Blood-letting is but a remedy, like digitalis or any other, and is to be treated as such. It has been potent for evil, and frightfully abused; but, in considering its use, we ought not to forget that our forefathers employed it, when now we resort to aconite and other sedatives, with whose action they were either entirely unacquainted or of which they understood but little. It is thus a remedy which they used, for which we possess substitutes which they did not know of. It seems but fair to bear this in mind when we take into account how often, and, according to our present ideas, how needlessly, they abstracted blood.

We repeat, however, that the whole question of antiphlogistics seems to us an entirely clinical one, to be solved only by observation, and not by reference to any views derived from the doctrine of cell-growth, of which we know so little. From its study, and by tracing out the natural history of disease, immense good may be expected. We shall learn more and more when to abstain from interfering, and when to assume an active part; when to sustain, and when to depress. The improvements in diagnosis have, and must enlarge our horizon more and more, and experimental observation of all remedies, the natural phases of diseases being perfectly understood, will teach us their exact value and application. But while, at present, we are not to be guided entirely by former experience, it is not right to eschew it entirely, and to declare against every depressing agent: it also is not inconsistent to admit that when an antiphlogistic plan of treatment was formerly so much more practiced, it was also really more called for. It is more creditable to the advance of knowledge, and to the reputation and honor of our profession, to uphold this view, and we cannot see that Dr. Bennett has advanced any facts which disprove it.

The agitation of this subject will doubtlessly lead to many valuable observations. It has already called into the lists Watson, Alison, Christison, and a number of less renowned champions, and is, for the most part, conducted with spirit and in a courteous manner. Here and there a little acrimony has been introduced; but Dr. Bennett possesses no ordinary skill, and it is better to meet so good a warrior, when in arms, in a friendly tournament, than to defy him to combat by touching his shield with the point of the lance.

The remaining sections of the work are taken up with the consideration of the diseases of separate systems, commencing with those of the *nervous system.* Among its diseases none is more important than *softening,* and here it is, also, where the microscope is of such signal advantage. Softening is usually taught to be dependent on acute or chronic inflammation, or on obliteration and disease of the arteries. Our author, from the analysis of numerous cases, has come to the conclusion that it may originate in six ways: from exudation which is infiltrated among the elementary nervous structures; from a mechanical breaking up of these structures by hemorrhagic extravasations, whether in mass or infiltrated in small isolated parts; from fatty degeneration of the nerve-cells, independent of exudation; from the mere imbibition of serum, which loosens the connection between the nerve-tubes and cells; from mechanical violence in exposing the nervous centres; and from putrefaction. It is impossible to distinguish these forms with certainty without the aid of the microscope, as the cases cited abundantly prove. We may, in truth, go further, and say that without its use, notwithstanding the presence of symptoms of softening during life, the very fact of the existence of this lesion may elude the close search of one whose eye is accustomed to look at morbid structures.

In cases of exudative or inflammatory softening the morbid mass contains numerous granules and granule-cells, which are seen coating the vessels or lying diffused between the tubes. These cases present also a marked contrast, clinically, to those in which the softening is not connected with inflammation. Thus, in twenty-four cases in which Dr. Bennett observed cerebral softening, eighteen presented the granular corpuscles, and were, therefore, presumed to be cases of exudative or inflammatory softening. In these persons well-marked symptoms invariably existed, such as loss of consciousness, preceded or followed by dullness of intellect, contraction and rigidity of the extremities, or paralysis. In the six cases of non-inflammatory softening there was no paralysis or contraction, and no dullness or disturbance of the intellect. In four cases, in which both lesions were present, symptoms could always be observed on the side opposite the inflammatory softening, but none existed opposite the non-inflammatory.

We are unacquainted, from its occurring but rarely pure, with the symptoms of fatty softening. Nor is it easy always to separate cases of chronic exudative softening from those resulting from hemorrhage. The suddenness of the attack in hemorrhage, and the more gradual signs of disturbance in the mental function, or in motion, in chronic inflammatory softening, are elements of the utmost diagnostic significance. The frequent complication, however, of chronic cerebral softening with hemorrhage, tends to obscure our landmarks, while, on the other hand, the exudative process occurring around the hemorrhage renders a correct diagnosis sometimes almost impossible. The researches of Dr. Todd have thrown some light on this difficult subject, and, it is to be hoped, will gradually clear it up.

Another point involving great difficulty in diagnosis is the distinction between acute *cerebritis* and *meningitis.* The only differences perceptible, when the substance of the brain is affected, are marked convulsions and paralysis, and a less degree of delirium, somnolence, and stupor. The latter is a disease of more frequent occurrence: Dr. Bennett records several interesting cases (Cases V. and VI.) of simple meningitis in tubercular persons. In the treatment of meningitis, as might have been expected from so zealous an adversary to the depressing treatment, antiphlogistics do not meet with much support. Nor has Dr. Bennett derived much advantage from the treatment of paralysis by strychnia. In cases of paraplegia, he believes it to have increased the patient's debility. Phosphorus, so much vaunted, did not succeed better. For the relief of functional nervous disorders the usual practice of administering tonics is firmly adhered to. We find in this part of the work several very instructive cases of poisoning by lead, opium, and hemlock, and also a useful classification of functional nervous disorder. There is but one point in it to which we would object, as likely, without explanation, to lead into error,—it embraces apoplexy. We suppose that this is meant to apply solely to those exceedingly rare and doubtful cases of nervous apoplexy, where post-mortem examination has failed to discover even any of the phenomena of congestion.

The diseases of the *liver*, involved at one time in such obscurity, are now better understood. This is certainly true of their pathology, and to a more limited extent of their therapeutics. The indiscriminate use of mercury has, nevertheless, retarded our knowledge of the use of other remedial agents in hepatic diseases. Iodine in chronic hepatitis has been of late years much employed; and we subjoin the following case as instructive, from proving not only its remedial effect even after mercury has failed, but also the possibility of entirely removing chronic enlargement of the liver and Bright's disease which accompanied it. The latter result was achieved by diuretics, which Dr. Bennett regards as the most certain of the remedies for this affection.

"HISTORY.—David Harper, aged thirty, painter, admitted into the clinical ward, February 18, 1852. Four months ago, was seized with diarrhœa and vomiting, which have continued more or less ever since. The liver was first observed to be enlarged in the beginning of December last, and it has gradually increased in size up to the present time. He has taken numerous remedies to check the diarrhœa and vomiting, but with little effect.

"SYMPTOMS ON ADMISSION.—On admission, the liver is found to extend from one inch below the right nipple above to within an inch and a half of the anterior superior space of the ilium below,—a depth of nine inches. From this point its margin could be felt ascending obliquely upward to the most depending portion of the ninth rib on the left side, crossing about an inch above the umbilicus. There is distinct fluctuation to be felt throughout the rest of the abdomen, indicating ascites. In the right lumbar region the enlarged liver is tender on pressure. The abdomen measures thirty-two and a half inches in circumference at its widest part. Spleen of normal size. Tongue moist, slightly loaded. There has been no vomiting for some days, but the diarrhœa is very severe. Says he has frequently passed blood by stool. Skin not jaundiced, but rather dry. Respiratory, circulatory, and other systems normal. ℞ Pil. Plumb. et Opii., xij. *Sumat unam ter indies.*

"PROGRESS OF THE CASE.—*March* 4.—Has had occasionally vomiting and diarrhœa since last report, for which he has been taking at times the naphtha mixture, morphia draughts, and gallic acid. To-day the urine is somewhat scanty, and slightly coagulable on the addition of heat and nitric acid; spec. grav. 1024. ℞ Acetatis Potassæ, ʒj; Sp. Æth. Nit., ʒij; Syr. Aurantii. ℥j; Aquæ, ℥v, M. Sumat ℥j ter indies. *March* 12.—To-day the urine was ascertained, with the microscope, to contain numerous casts of the tubes and isolated epithelial cells loaded with fatty granules. The vomiting and diarrhœa continue. *Habeat suppositorium quaque 8va hora. April* 6.—The diarrhœa was for a few days somewhat checked by the suppositories, but gradually returned, and is now very severe; the bowels having been opened twelve times yesterday. The urine has continued albuminous, and loaded with desquamative casts and fatty tubes. To-day its spec. grav. is 1007. There is now great debility, and occasional stupor and drowsiness. *May* 12.—The drowsiness has disappeared. For the last few days has been taking ʒj of the potass. bitart. with the mixture of acetate of potash and nitric ether, and he now passes a larger amount of urine, which is free of tubular casts. The abdomen is less tense. About the middle of May the vomiting and diarrhœa first abated, and was soon after checked. In August his health was so much improved that he was allowed to go out of the house for the benefit of air and exercise. He was readmitted *September* 13, having enjoyed tolerable health in the interval, although the hepatic swelling is about the same size. He was now ordered, ℞ Hydrarg. Proto Iodidi, gr. vj; Pulv. Opii, gr. ij; Ext. Taraxaci, ʒss; Conserv. Rosarum, gr. v, fiant pil. xx. Sumat 1 ter indies. These pills on the 20th produced salivation, when they were discontinued, and an astringent gargle ordered. The abdomen now measures thirty-six inches in its broadest circumference. *Oct.* 25.—Complains of oppression on walking, of shooting pains through the chest and abdomen. Ascites seems once more to be increasing. *Tr. Iodini to be painted over the abdominal surface. Nov.* 21.—Since last report the liver has greatly diminished in size, and his complaints have ceased. The urine presents a slight hazy albuminous appearance on the addition of heat and nitric acid, but is voided in natural quantity. *Dec.* 13.—The liver is now so reduced in size that its lower margin is only two inches below the false ribs in front, and one inch on the right side. All his functions are apparently healthy, the urine healthy, and his strength appears perfectly re-established.

"He has since been seen by the clerks walking about the town, and has informed them that he is quite well and carries on his occupation without any inconvenience."

In the chapter on diseases of the *digestive* system we find mention made of the decoction of the unripe fruit of the Indian Bael, better known as the Bengal Quince, as an exceedingly valuable astringent in the treatment of chronic diarrhœa and dysentery. The decoction must be used fresh, and is prepared by simmering two ounces of the unripe fruit in a pint of water down to a fourth, of which from one to three table-spoonsful constitute a dose. We also find a most instructive case, in a diagnostic point of view, of cancer of various abdominal organs producing all the symptoms of acute peritonitis, including great tenderness, with distension of the abdomen, and fever; the fluid and the tympanitis masked the nodular swellings found after death.

In the treatment of *pericarditis*, mercury does not meet, in the hands of Dr. Bennett, with the favor usually accorded to it. Since the days in which Graves expressed his conviction that all our efforts must fail unless a speedy mercurialization be produced, this remedy has been considered as the most important in the removal of pericardial inflammations. Its efficacy was questioned by the late Dr. Taylor from an analysis of forty cases. Dr. Bennett adopts his conclusions, and states that, after having administered it in many cases, he could never satisfy himself that it had the slightest influence in forwarding or modifying the natural changes which occur. Yet few of the cases he has recorded are pure, most being complicated with other affections, many with disease of the lung or heart. It may, however, be very justly urged that uncomplicated pericarditis is among the rarest of clinical phenomena. In sixty-two cases recorded by Bamberger (Virchow's Archiv., vol. ix.) only three cases of pure pericarditis are met with; and Dr. Bennett might cite this very paper as confirming his views, since of twenty-nine cases treated without a grain of mercury, only two died, one a woman of eighty-nine years of age, and the other was complicated with typhoid pneumonia. This same author reports the case of a hatter, the subject of mercurial tremor, who, notwithstanding his system was saturated with the drug, was seized with acute pericarditis.

The same objection to mercury, and indeed to any antiphlogistic treatment, is visible in Dr. Bennett's treatment of acute inflammations of the *respiratory* organs. Calomel in acute pleurisy and in acute pneumonia he believes to be worse than useless. His treatment is to further the natural progress of the disease by securing rest, avoiding lowering remedies, and administering salines and diuretics to favor excretion of the morbid products, and wine and nutrients, should the pulse be weak; local pain to be relieved by warm fomentations or poultices. If statistics can determine the superiority of this plan of treatment, they certainly would do so by overwhelming numbers; for we find but a mortality of one case in twenty-three, whereas the mortality by extensive blood-letting is nearly one in three, (Louis;) by large doses of tartar emetic one in five, (Rasori;) by moderate bleeding (Laennec and Grisolle) one in seven to ten.

The progress of a case of pneumonia may be for the most part accurately told by the absence or presence of chlorides in the urine. They disappear during its progress, they reappear at its cessation in company with the returning crepitation. A case of great interest, as bearing on this point, is the one cited, p. 638. It was that of a man, aged twenty-six, laboring under typhus fever, in whom on the eleventh day the chlorides disappeared from the urine. This led to an examination of the chest, when the signs of pneumonia were detected in the lower half of the right lung. On the fourteenth day the chlorides reappeared, and the pneumonic signs diminished.

In the treatment of the chronic diseases of the lungs, Dr. Bennett, as is well known, is an ardent supporter of cod-liver oil. The local treatment of chronic bronchitis by nitrate of silver is one he encourages.

*Leucocythemia*, or, as Virchow calls it, *leucæmia*, is a disease the knowledge of which dates from 1845. We do not wish to enter the arena for or against either of the rival professors who claim its discovery; we believe that our thanks are due to both. Bennett, as the date of his publication shows, was the first to draw attention and give an impetus to the whole subject; but Virchow just as undoubtedly was the first who understood the true nature of the cases. Bennett showed the presence of corpuscles in the blood unconnected with phlebitis and associated with enlargement of the spleen; but Virchow demonstrated them to be blood corpuscles, and proved that they might occur as dependent upon enlargements of the lymphatic glands, proposing, at the same time, the name of leucæmia, or white blood, for the disease. Not to enter further into this discussion,* we would say that Dr. Bennett has contributed most to the clinical

* If any of our readers should wish to inform themselves at length as to the facts relative to the question at issue, we refer them to Dr. Bennett's statements in the work under review; to Kolliker's statement in the Edinburgh Month. Jour. of Med. Science, 1854; to the Dublin Medical Press; Vidal, Gazette Hebdomadaire, 1855; and to Virchow, in the seventh volume of his Archiv. fur Path. Anatomie.

study of the subject, and that the result has been the knowledge of a disease whose anatomical elements consist in an enlargement, usually a simple hypertrophy, of the spleen, a hypertrophy of the liver, and an enlargement of the mesentric glands, and sometimes of the thyroid gland and supra-renal capsules; while its symptoms are a preponderance of white corpuscles in the blood, readily seen by pricking the finger, a pale complexion, cachectic appearance, a tendency to diarrhœa and dyspnœa, and usually the physical signs of enlargement of one or more of the organs indicated, and a progressive diminution of strength. This latter symptom is unfortunately the rule. Virchow says, "as yet no well-proved case of cure is known," and Bennett does not make a more encouraging remark when he states that "with regard to treatment, nothing that I have yet tried has appeared to be of the slightest service in well-marked cases with distinct glandular enlargements. Iron, quinine, chloride of potassium, hydriodate of potassa, and a variety of remedies given internally, with tincture of iodine applied externally, have been of no avail. The chief indications in advanced cases, however, will be found to be furnished by accidental complications, the most common of which are diarrhœa and epistaxis, which require astringents, combined with tonics, nutrients, and stimulants, to support the vital powers."

Nor can the pathology of the disease be regarded as entirely settled, since it involves the whole question of the formation of the blood corpuscles. This much is certain, that the lymphatic and other ductless glands have a large share in their formation. But it has been supposed probable that the corpuscles are formed in the blood itself. Dr. Bennett's theory is that the colored blood corpuscles are free nuclei, sometimes coming as such from the glands, at others developed within colorless cells. These latter, the white corpuscles, are always derived from the lymphatic glandular system. In certain hypertrophies of this system the cell-elements are multiplied to an unusual extent, and find their way into the blood, constituting an increase in the number of its colorless cells. A corresponding diminution in the formation of free nuclei, and consequently of colored corpuscles, occurs. This is leucocythemia; and is also the best theory which the present state of knowledge of the subject offers.

Dr. Bennett explains the views with reference to the formation of blood corpuscles at some length. If we were disposed to be critical, we should say at too great a length. In a future edition we hope to see this and the controversy about the history of the subject somewhat abridged; and, considering these are clinical lectures, a less meagre and more satisfactory account of the symptoms of this strange disease substituted.

But we must here take leave of our author. We had marked for further consideration an account of his treatment of rheumatism by nitrate of potassa; of scabies without sulphur ointment; a substitute for mercurial plaster in smallpox, in the common calamine saturated with olive-oil; and a most astonishing case, which must have been a perfect *bonne bouche* for any medical student eager of wonderful cases, a person presenting a thoracic aneurism; an abdominal aneurism; the treatment of aneurism by Valsava's method; acute passing into chronic pleurisy; gradually increasing and at length complete paraplegia; and the most rapid death by aconite on record. We had marked these, and we might have marked many more subjects, without having exhausted the interest or the originality of this volume. We have indeed rarely met with a more thoughtful work; nor do we believe that there is one extant which in many respects better reflects the present advanced state of medicine. It is a pioneer treatise from one who has brought into a volume, abounding with interesting matter, the research and the illustration furnished by modern science, and has best urged its claims He may have arrived at conclusions which are premature or debatable, but the enlightened skepticism he displays, (although it would seem to us to go here and there too far,) and the thorough devotion to his subject, are worthy of the highest praise. The book is not merely one of reference, but one to reflect over. As a text-book to a student it may not be the best, as it is too unequal and incomplete; but as a book to take down from our shelves and frame our observations on, and learn how to throw light on daily practice, it is without a rival. The author tells us that when he finds time he will devote himself to the consideration of the pathology and causes of inflammation; we, his brethren on this side of the Atlantic, bid him God-speed in this difficult task.

